# Process Optimization of Silver Nanoparticle Synthesis and Its Application in Mercury Detection

**DOI:** 10.3390/mi12091123

**Published:** 2021-09-18

**Authors:** Lung-Ming Fu, Jia-Hong Hsu, Ming-Kuei Shih, Chang-Wei Hsieh, Wei-Jhong Ju, Yu-Wei Chen, Bao-Hong Lee, Chih-Yao Hou

**Affiliations:** 1 Department of Engineering Science, National Cheng Kung University, Tainan 701, Taiwan; loudyfu@mail.ncku.edu.tw (L.-M.F.); david6767@gmail.com (W.-J.J.); 2 Department of Seafood Science, National Kaohsiung University of Science and Technology, Kaohsiung 811, Taiwan; F107176106@nkust.edu.tw; 3 Graduate Institute of Food Culture and Innovation, National Kaohsiung University of Hospitality and Tourism, Kaohsiung 812, Taiwan; mkshih@mail.nkuht.edu.tw; 4 Department of Food Science and Biotechnology, National Chung Hsing University, Taichung 402, Taiwan; welson@nchu.edu.tw; 5 Department of Medical Research, China Medical University Hospital, Taichung 404, Taiwan; 6 Department of Medicine, Chang Gung University, Linkow 333, Taiwan; naosa720928@gmail.com; 7 Department of Horticulture, National Chiayi University, Chiayi 600355, Taiwan; bhlee@mail.ncyu.edu.tw

**Keywords:** silver nanoparticles (AgNPs), nanoparticle size analysis, process optimization, citrate, sodium borohydride, mercury

## Abstract

Silver nanoparticles (AgNPs) have stable reactivity and excellent optical absorption properties. They can be applied in various industries, such as environmental protection, biochemical engineering, and analyte monitoring. However, synthesizing AgNPs and determining their appropriate dosage as a coloring substance are difficult tasks. In this study, to optimize the process of AgNP synthesis and obtain a simple detection method for trace mercury in the environment, we evaluate several factors—including the reagent addition sequence, reaction temperature, reaction time, the pH of the solution, and reagent concentration—considering the color intensity and purity of AgNPs as the reaction optimization criteria. The optimal process for AgNP synthesis is as follows: Mix 10 mM of silver nitrate with trisodium citrate in a hot water bath for 10 min; then, add 10 mM of sodium borohydride to produce the AgNPs and keep stirring for 2 h; finally, adjust the pH to 12 to obtain the most stable products. For AgNP-based mercury detection, the calibration curve of mercury over the concentration range of 0.1–2 ppb exhibits good linearity (R^2^ > 0.99). This study provides a stable and excellent AgNP synthesis technique that can improve various applications involving AgNP-mediated reactions and has the potential to be developed as an alternative to using expensive detection equipment and to be applied for the detection of mercury in food.

## 1. Introduction

The application of nanotechnology has become increasingly well-developed and widespread, contributing to the sustainable competitiveness and continuous growth of several industrial applications [1]. Nanoparticles (NPs) have valuable functions owing to their unique chemical and physical properties [2]; consequently, they have been applied in various fields such as medicine, biotechnology, materials science, and energy. Nanostructures are the critical factors of all applications involving nanotechnology in nature, and the properties of NPs are determined by their size [3]. Metal NPs contain a range of materials, from a few to several metal atoms whose surfaces are stabilized by ligands, surfactants, polymers, or dendrimers. Metal NPs play a vital role in catalysis because of their nanoscale size and their ability to improve the efficiency of heterogeneous catalysis by mimicking metal surface activation [4,5]. This also applies to homogeneous catalysis, as there exists a wide range of tiny metal clusters and bulky metal clusters, which are all eventually called colloids or NPs [6].

The production of NPs generally involves physical and chemical processes. Both are applicable to AgNP synthesis. Chemical reduction, which uses organic and inorganic reducing agents, is the most common method for the synthesis of AgNPs. This technique involves a single process to generate a colored silver solution, as the metal surface contains free electrons in the conduction band and positively charged nuclei. Then, long-lived clusters of silver are formed, and the synthesis of AgNPs is confirmed [7]. Generally, the one-pot method of AgNO_3_ reduction involves different reducing agents, such as trisodium citrate, ascorbate [8,9], sodium borohydride (NaBH_4_) [7,10,11], elemental hydrogen, polyol process, N,N-dimethylformamide [7,12], ascorbic acid, poly(ethylene glycol)-block copolymers [13], hydrazine, and/or ammonium formate [7]. They are applied to reduce silver ions (Ag^+^) in aqueous or non-aqueous solutions [12]. The chemical reduction method requires three reactants, namely, a silver precursor, a capping agent, and a reducing agent. The reducing agent reduces the silver precursor to the state of silver particles, while the capping agent covers the outer layer of silver particles, making them less likely to aggregate. The morphology of the AgNPs can also be changed by adjusting the concentration and dosage of the reagents, among other factors.

Because of the increased risk of mercury contamination, more and more environmental, food, and health problems have arisen. Therefore, developing a nano-sensing technology that can detect mercury ions at a level as low as 1 ppb is necessary in order to provide the information for mercury pollution detection [14]. In recent years, the number of works focused on developing novel nanoparticle-based sensors for mercury detection has increased, motivated mainly by the need for low-cost portable devices capable of providing a fast and reliable analytical response, thus contributing to analytical decentralization. Botasini et al. reported that the sample matrix highly affects the response of nanoparticle-based sensors [15]. The developed analytical nanosystems may fail in actual samples because of the adverse incidence of ionic strength and the presence of exchangeable ligands [16,17]. Therefore, optimizing and establishing a methodology of mercury detection in silver nanoparticles is very worthy of study.

It is well-known that techniques used for the sensitive and selective detection of Hg^2+^ include cold vapor atomic absorption spectrometry (CVAAS), cold vapor atomic fluorescence spectrometry (CVAFS), and inductively coupled plasma mass spectrometry (ICP-MS). However, these high-performance techniques often require expensive equipment and technical expertise for sample preparation [18,19,20,21]. Because of these shortcomings, it is advantageous to use silver nanoparticles (AgNPs) as a colorimetric sensor to provide a low-cost and rapid method for the detection of mercury in the environment. These analytical methods convert the analyte concentration to a different color, which makes simple on-site qualitative and quantitative applications through colorimetric or visual inspection possible. Therefore, the colorimetric method using AgNPs and AuNPs is a promising tool for monitoring mercury content, especially in highly polluted areas [22,23,24,25]. The World Health Organization (WHO) has stated in the ASSURED Challenge [19] that access to equipment should not limit the diagnostic test performance, especially in developing countries and other resource-limited regions. The quantitative and quantitative detections of all chemical hazards require a fast, simple, and inexpensive approach. Although AgNPs can be applied in various industries because of their stable reactivity and excellent optical absorption properties, synthesizing AgNPs and determining their appropriate dosage as a coloring substance are difficult tasks.

To optimize the process of AgNP synthesis and obtain a simple detection method for trace mercury in the environment, we evaluated several factors—such as the reagent addition sequence, reaction temperature, reaction time, the pH of the solution, and reagent concentration—while considering the color intensity and purity of AgNPs as the reaction optimization criteria. This research aims to provide the best AgNP preparation method for academic applications, and it is expected that in the future this excellent chemical coloration method can be combined with a suitable, convenient, and low-cost detection mode to detect mercury contamination in food.

## 2. Materials and Methods

### 2.1. Experimental Methods

#### 2.1.1. Exploration of the Optimal AgNP Formation Conditions

The AgNPs were generated by a simple and easy-to-use chemical reduction method using a silver precursor, capping agent, and reducing agent [26]. Moreover, we investigated the optimal AgNP formation conditions by focusing on five influencing factors of the reaction: the reagent addition sequence, reaction temperature, reaction time, reagent concentration, and pH.

The AgNPs were synthesized using three reagents: silver nitrate, trisodium citrate, and sodium borohydride. The addition sequence of these reactants affects the morphology of the generated AgNPs. Therefore, the most appropriate reaction conditions were identified by comparing different reagent addition sequences. The experimental design started with the common step—adding silver nitrate and trisodium citrate to deionized water and mixing for 10 min. This is followed by one of three addition sequences: AgNPs (I)—Add sodium borohydride to produce AgNPs and keep stirring for 2 h; AgNPs (II)—Add trisodium citrate to produce AgNPs and keep stirring for 2 h; AgNPs (III)—Add silver nitrate to produce AgNPs and keep stirring for 2 h. The absorbance values of the above three sets of AgNPs generated by different reagent addition sequences were measured using an enzyme immunoassay analyzer (BioTek, Epoch 2, Winooski, VT, USA) in the wavelength range of 300–600 nm, and the differences among the sets were compared.

During the generation of AgNPs, the reaction temperature affects the production rate and morphology of the NPs. Therefore, the AgNP generation reaction needs to be conducted at different temperatures. As the reaction rate increases, the reactants are consumed faster; hence, reactant depletion occurs, leading to the formation of smaller nanoparticles and narrow size distribution at higher temperatures [27]. We conducted the experiments in a hot water bath (100 °C) and an ice bath (−1 °C). When the AgNPs are generated, the mixing time of the reagents affects the final morphology of the AgNPs. The AgNP formation process was divided into two steps: First, the reaction vial was placed in a hot water bath, and deionized water, silver nitrate, and trisodium citrate were added to the reaction vial and mixed for 0–60 min; then, sodium borohydride was added to produce AgNPs, and the reaction was continued for 120 min. A total of 36 sets of AgNPs were produced. As previously described, the absorbance values of the 36 sets of AgNPs fabricated with different combinations of the two time parameters were measured and compared.

The pH value of the solution is a critical influencing factor of chemical reactions and is also essential for the production of AgNPs. This study explored the impact of the pH of the solution on the morphology of the AgNPs. The experimental design was as follows: Deionized water, silver nitrate, and trisodium citrate were added to the reaction vial in a hot water bath and mixed for 10 min.; then, sodium borohydride was added to produce AgNPs, and the reaction was continued for 2 h; at the end of the reaction, the pH value of the solution was measured using a pH meter (SUNTEX, SP-2500, New Taipei, Taiwan), and the solution was adjusted to be acidic or alkaline using citric acid or sodium hydroxide. The absorbance values of the AgNPs from the reaction vial after pH adjustment were measured and compared, as previously described.

The concentrations of the silver nitrate, trisodium citrate, and sodium borohydride reagents may affect the generation of AgNPs and their concentration. The design of the related experiment in this study was as follows: The three reagents—silver nitrate, trisodium citrate, and sodium borohydride—were prepared at the concentrations of 0.1, 1, and 10 mM for the AgNP synthesis reaction. A total of 27 sets of AgNP solutions with different combinations of reagent concentrations were obtained. The absorbance values of the above 27 sets of AgNPs were measured and compared, as previously described.

#### 2.1.2. AgNP Characterization

After the preparation of AgNPs, the absorption wavelength range of the AgNPs was preliminarily determined by spectroscopy. First, 200 μL of AgNP solution was added to each cuvette in a 96-well sample holder, and the samples were scanned using an enzyme immunoassay analyzer (BioTek, Epoch 2, Winooski, VT, USA) with the visible spectrum in the wavelength range of 300–600 nm.

The size of an NP is typically defined as 100–2500 nm, whereas the size range of AgNPs is defined as 1–100 nm [28]. The diameter of the generated AgNPs was measured using a nanoparticle size analyzer (Brookhaven, 90Plus, Holtsville, NY, USA) in order to confirm that the silver produced in our experiments was AgNPs, and the average particle size was determined simultaneously. We observed the shape and dispersion pattern of the AgNPs and also measured their size. Accordingly, 10 μL of AgNP solution was dropped onto a carbon-coated copper grid and dried at room temperature (25–28 °C), and the dispersion pattern, particle shape, and diameter of the AgNPs were examined using a Transmission Electron Microscope (TEM) (JEOL, JEM-3010, Peabody, MA, USA).

#### 2.1.3. Silver Nanoparticle Spectroscopy for Mercury Detection

Through the chemical coloring method, the reaction between AgNPs and mercury leads to a color change, such that the mercury content in a sample can be quantified. In the coloring reaction, the volume ratio of the coloring agent and the analyte may affect the detection result. We tested the volume ratios of the AgNP solution to mercury standard of 1:0.01, 1:0.1, 1:0.5, 1:1, and 1:2, and the absorbance values were measured at a wavelength of 400 nm. The mercury standards were prepared with 0, 0.001, 0.01, 0.1, and 1 N nitric acid, respectively. Mercury solutions with a concentration of 0.1, 1, or 10 ppb were reacted with AgNPs for coloring, and the absorbance value of the solution was measured at a wavelength of 400 nm. From the results of the above experiments, we identified the optimal reaction conditions, constructed a calibration curve for 0.1–2 ppb mercury, and measured the absorbance values of the samples at a wavelength of 400 nm after the coloring reaction with the AgNPs.

In the false-positive tests, we used metal ions—including sodium, iron, copper, lead, nickel, and magnesium ions—contained in sewage as interferents and deionized water as a blank control to react with the AgNPs. The absorbance values of the samples were measured using an enzyme immunoassay analyzer at a wavelength of 400 nm.

#### 2.1.4. Cold Vapor Atomic Absorption Spectrometry for Mercury Detection

We referred to the method provided by the American Public Health Association [29] and applied minor modifications for comparison with the conventional method. We used cold vapor atomic absorption spectroscopy to determine the mercury content, with hydrochloric acid and sodium borohydride as the reaction reagents. A mercury standard was added to the reaction vial using a peristaltic liquid pump and mixed. The mercury atoms were vaporized by the cold vapor generated by the reaction between hydrochloric acid and sodium borohydride and sent to the absorption tube. Then, the absorbance value of the mercury standard was determined using a cold vapor atomic absorption spectrometer (Hitachi, Z-5300, Berkshire, UK) at a wavelength of 253.7 nm. A calibration curve was constructed using the measured absorbance values.

## 3. Results

### 3.1. Generation of AgNPs

**Figure 1:**
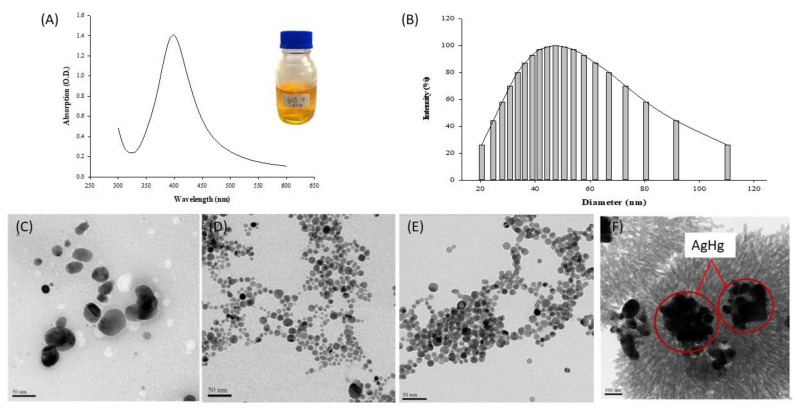
Generation of AgNPs: (**A**) UV–Vis absorption spectrum of silver nanoparticles (AgNPs) with λ = 300–600 nm, the central peak is concentrated at 350–450 nm. The silver nanoparticle colloid color can be seen to be bright yellow; (**B**) Nanoparticle Size Analyzer (NSA) size analysis of silver nanoparticles (AgNPs). The AgNP particle size was measured mainly at 47 nm; (**C**) TEM image of silver nanoparticles (AgNPs) synthesized by silver nitrate, trisodium citrate, and sodium borohydride. TEM image of silver nanoparticles (AgNPs): silver nanoparticles were synthesized when sodium borohydride was added and stirring was stopped immediately (**D**), and after 60 min (**E**); and (**F**) TEM image of Ag amalgam crystals (AgHg).

The AgNPs produced in our experiments were confirmed by absorbance measurements in the range of 300–600 nm. The absorption peaks of the AgNPs were identified, and the results are shown in Figure 1A. The generated AgNP solution had a bright yellow color. The absorption peaks of the AgNPs formed by chemical reduction using silver nitrate, trisodium citrate, and sodium borohydride were concentrated within 350–450 nm, and the maximum absorption was detected at 400 nm. In the literature, with silver nitrate as the silver precursor, the absorption peaks of AgNPs produced with different capping and reducing agents have generally been reported in the wavelength range of approximately 400 nm [30,31]. The dominant wavelength range varies with the use of different capping and reducing agents for AgNP generation. For example, AgNPs fabricated by a green synthesis method using plant extracts had absorption peaks in the 407–450 nm wavelength. The literature has also indicated that the color of the AgNP colloidal solution can serve as a preliminary judgment criterion for the completion of AgNP formation in the reduction reaction, where a yellow color indicates that the AgNP synthesis process has been completed [32].

The particle size and average dispersion of the generated AgNPs were measured using a nanoparticle size analyzer (Brookhaven, 90Plus, Holtsville, NY, USA). The AgNP colloidal solution was injected into a 1 cm cuvette and measured 10 times by scanning, and the data are plotted in Figure 1B. The diameter of the AgNPs synthesized by the chemical reduction method was in the range of 20–110 nm, and the size distribution intensity was the greatest at 47.4 nm, indicating that the average dispersion of AgNPs was the highest in this range. Previous AgNP synthesis studies using the chemical reduction method have shown that the concentrations of the reducing and capping agents and their volume ratio can affect the morphology of the generated AgNPs. As the ratio of the capping agent to the reducing agent increased from low to high, the final diameter of the AgNPs gradually increased. When the ratio of the two reagents was 0.005:1, the average size of the synthesized particles was 10.1 nm. With an addition ratio of 1:1, the average NP diameter was 46.1 nm. The reaction volume of the capping agent in AgNP synthesis affected the final size of the NPs [32,33].

The morphology of the generated AgNPs was observed using a TEM (JEOL, JEM-3010, Peabody, MA, USA), and the size of the NPs was confirmed simultaneously. The NP size was distributed in a wide range. As revealed in Figure 1D,E, although the size of the AgNPs had a bimodal distribution, with small and large particles, they were all round. The measured particle size was in the range of 11.1–88 nm, and the size with the highest frequency was approximately 43.2 nm, similar to the results obtained using a nanoparticle size analyzer [33]. The AgNPs formed at different time points (0 and 60 min) after the reaction with silver nitrate, trisodium citrate, and sodium borohydride were photographed and observed. The AgNPs generated at 0 min after the reaction (Figure 1D) were uneven in shape, whereas their size was more consistent than that of the AgNPs generated at 60 min after the reaction (Figure 1E). The particle size measurements revealed that the diameter of the AgNPs at 0 min after the reaction was in the range of 10.4–29.1 nm, with an average of 16.3 nm, whereas the AgNPs at 60 min after the reaction had sizes in the range of 6–35.6 nm, with an average of 13.4 nm [28].

Figure 1F is a TEM image demonstrating (A) the AgNPs not bound with mercury and (B) those bound with mercury in amalgam crystals (the red cycle). The AgNP-based mercury detection reaction produces a metallic alloy called amalgam. In this reaction, mercury ions are adsorbed onto the surface of AgNPs, because of the latter’s vibration characteristics, such that detection can be realized [34]. In the present study, the AgNPs generated by chemical reduction were reacted with mercury; then, the pattern of the two metal ions bound together was observed using a TEM (JEOL, JEM-3010, Peabody, MA, USA). As AgNPs have a single-grain structure with a tiny size, when mercury is added to the AgNP colloidal solution, the AgNPs adsorb mercury ions to form amalgam crystals, and their particle size will increase accordingly. Moreover, mercury ions not only become adsorbed onto single silver particles but also bind with groups of silver particles into aggregates. Therefore, the size of the AgNPs increased from 43.2 nm to 160–270 nm [31,35].

### 3.2. Influencing Factors on AgNP Synthesis

#### 3.2.1. Reagent Addition Sequence and Temperature Conditions

**Figure 2:**
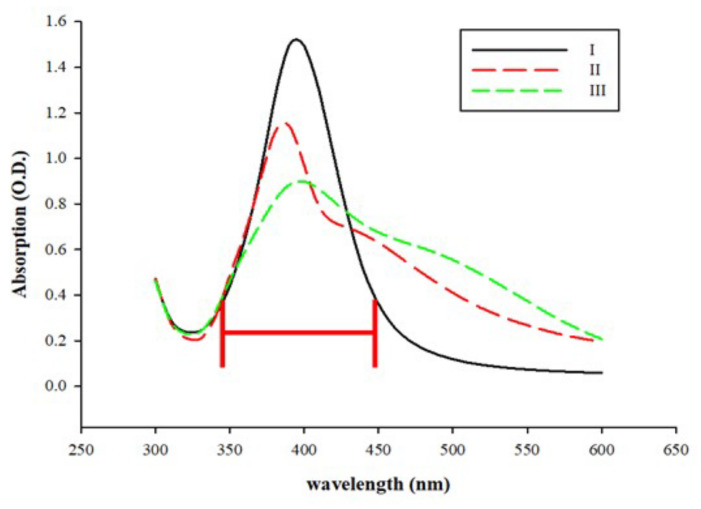
Effects of reagent addition sequence on the formation of AgNPs: UV–Vis spectrum (λ = 300–600 nm) of silver nanoparticles. Effect of reagent addition order on silver nanoparticles synthesized by silver nitrate (AgNO_3_), trisodium citrate (SC), and sodium borohydride (NaBH_4_). I: AgNO_3_ + SC mix 5 min, add NaBH_4_ then mix for 2 h. II: AgNO_3_ + NaBH_4_ mix 5 min, add SC, then mix for 2 h. III: SC + NaBH_4_ mix 5 min, add AgNO_3_, then mix for 2 h.

The AgNPs were synthesized using three chemical reagents (silver nitrate, trisodium citrate, and sodium borohydride) with different reagent addition sequences. The results are illustrated in Figure 2. The absorption spectrum of the AgNPs (I) showed a peak at 400 nm. Only if silver nitrate was mixed with the capping agent (trisodium citrate) before being reduced by the reducing agent (sodium borohydride) did the synthesized AgNPs maintain a desirable particle size. In the AgNP fabrication reaction, each environmental factor and reaction condition affects the final morphology of the NPs [36]. When the color of the AgNP colloidal solution changes from bright to turbid dark, it indicates that the AgNPs have agglomerated, and the measured particle size will continually increase [33]. As shown in Figure 2, among the three reagent addition sequences, sequence (I) was the optimum, and the corresponding absorption peak was more concentrated around 400 nm, without significant spectral shifts. The reason for this is that the silver nitrate had homogenously mixed with the capping agent before reacting with the reducing agent; hence, the generated NPs were more stable [37].

**Figure A1:**
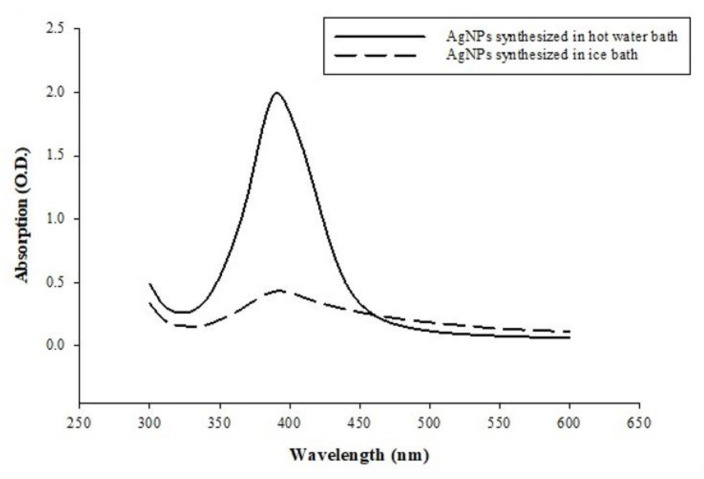
Effects of reaction temperature on the formation of AgNPs: UV–Vis spectrum (λ = 300–600 nm) of silver nanoparticles. Silver nanoparticles synthesized by silver nitrate, trisodium citrate, and sodium borohydride at different temperatures.

In generating silver nanoparticles, the temperature during the reaction affects the rate and type. The optical properties of silver nanoparticles are related to the excitation of plasmon resonance or inter-band transition, particularly the size effect. The UV spectroscopy method can be used to track the size evolution of silver nanoparticles based on localized surface plasmon resonance bands exhibited at different wavelengths. These peaks are characteristic plasmon bands for silver nanoparticles [38]. Mohammed Fayaz et al. reported that silver nanoparticles synthesized at 27 °C show spherical and occasionally rod-like silver nanoparticles ranging from 10–40 nm. Lower temperatures of 10 °C lead to the formation of silver nanoplates, with sizes ranging from 80 to 100 nm [39]. As the reaction rate increases, the reactants are consumed faster; hence, reactant depletion occurs, forming smaller nanoparticles and narrow size distribution at higher temperatures [40]. Therefore, in this study, the formation reaction of silver nanoparticles was compared with the use of a hot water bath (100 °C) and ice bath (−1 °C), confirming that high temperature maintains the characteristics of such particles and provides better observation conditions of light absorption and color development. The results are shown in Figure A1, where the dashed line represents the absorption spectrum of the AgNPs produced in the ice bath. The absorbance values on this line are generally lower than those of the AgNPs synthesized in the hot water bath.

Among the many factors that affect the characterization of silver nanoparticles, temperature changes affect the size and shape of silver nanoparticles, directly causing monodispersity and alterations in the spectral response. For example, Mock et al. reported that specific geometrical shapes lead to distinct spectral responses. In addition, inducing subtle changes in the particle morphology by heating causes a shift in the spectra of individual particles. This provides a simple means of tuning the spectral response to a desired optical wavelength [41]. Mohammed Fayaz et al. reported the effect of temperature on controlling the size of silver nanoparticles in the aqueous-based biosynthesis. An increase in reaction temperature led to a decrease in the size of silver nanoparticles and an increase in monodispersity [39]. Therefore, the temperature (as reflected in Figure A1) affects the production of AgNPs, consistent with previous reports [39,41].

#### 3.2.2. Different Reaction Times to Generate AgNPs

**Figure 3:**
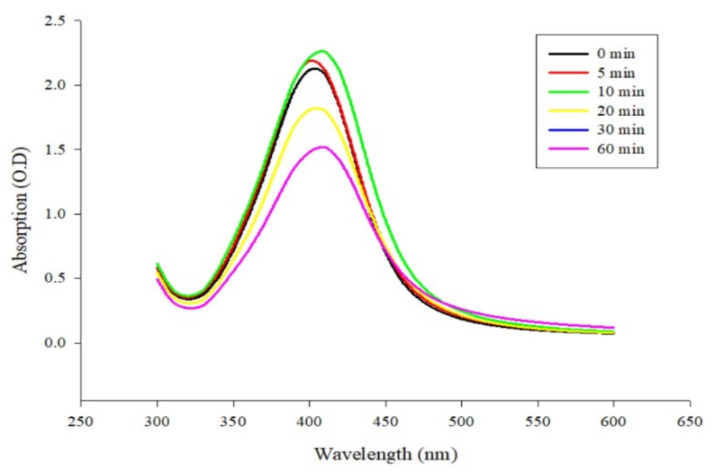
The ultraviolet-visible absorption spectrum of silver nanoparticles (AgNPs) was synthesized by reducing silver ions with trisodium citrate (SC) for 0 to 60 min (0, 5, 10, 20, 30, and 60 min). Then sodium borohydride was added to the silver nitrate-trisodium citrate solution and kept stirring for 120 min.

The absorption spectra in the wavelength (λ) range of 300–600 nm measured at different time points are illustrated in Figure 3. In the experiments, sodium borohydride was added to the reaction vial after silver nitrate had been mixed with trisodium citrate for various times (0, 5, 10, 20, 30, and 60 min), as shown in Figure 3, in order to produce AgNPs by reduction, and the solution was continuously stirred for 120 min. The absorbance values of the generated AgNPs increased with the stirring time. AgNPs started to form immediately after the addition of sodium borohydride. The shape of the initially produced AgNPs was relatively unstable, and their size was relatively large. As the stirring time increased, the NPs were more evenly dispersed and more stable [37,40,41]. Therefore, the best test condition was to keep stirring the reaction solution containing the silver nanoparticles for 120 min after incubating with sodium borohydride for 10 min.

#### 3.2.3. The Influence of Stirring Time and pH Value on AgNPs

**Figure 4:**
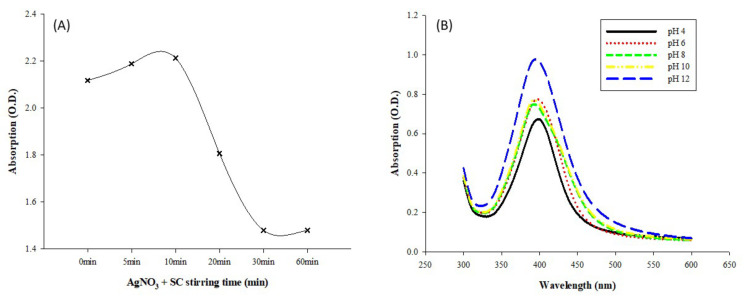
Effects of mixing time and pH on the generation of AgNPs: (**A**) UV–Vis absorption spectrum (λ = 300–600 nm) of silver nanoparticles synthesized by silver nitrate, trisodium citrate, and sodium borohydride at different synthesizing times; and (**B**) UV–Vis absorption spectrum (λ = 300–600 nm) of silver nanoparticles synthesized by silver nitrate, trisodium citrate, and sodium borohydride in different pH value solutions (pH 2–12).

In the first stage, silver nitrate and trisodium citrate were mixed for 0–60 min and then sodium borohydride was added in the second stage to produce AgNPs. The mixing time in the first stage also impacted the finally generated AgNPs, as revealed in Figure 4A. Comparing all the synthesized AgNPs at 120 min after the addition of sodium borohydride, we concluded that a reagent mixing time of 10 min in the first stage would be the optimal condition for the subsequent AgNP production. When the mixing time was less than 10 min, silver nitrate and trisodium citrate were not well-mixed and dispersed in the solution. However, after 10 min, the mixing time was too long, and the reaction reagents could be lost. Therefore, the optimal reaction time consisted of a 10 min mixing period in the first stage, followed by a 120 min AgNP generation period in the second stage.

To investigate the influence of pH on AgNPs, we adjusted the pH value of the generated AgNP solution from 4–12 using citric acid and sodium hydroxide. The determined absorption spectra in the wavelength range of 300–600 nm are shown in Figure 4B. The pH of the initially generated AgNP colloidal solution was 8. The measured absorbance values of the solution generally decreased when the solution was adjusted to an acidic environment. In contrast, the absorbance values increased when the AgNPs were in an alkaline environment. This is because AgNPs are unstable under acidic or neutral conditions, but the NPs favor an alkaline environment. Previous studies have argued that the pH value has significant impacts on AgNPs by changing their surface charges and indirectly affecting the stability and aggregation of the particles [42,43]. The literature has indicated that AgNPs are very unstable in acidic or neutral environments. Under such conditions, AgNPs have denser surface charges, making their aggregation more severe and their size larger. The decreased absorbance values of AgNPs may also be caused by the displacement of absorption peaks in surface plasmon resonance spectroscopy [30,41].

#### 3.2.4. The Influence of Reagent Concentration on AgNP Synthesis

**Figure 5:**
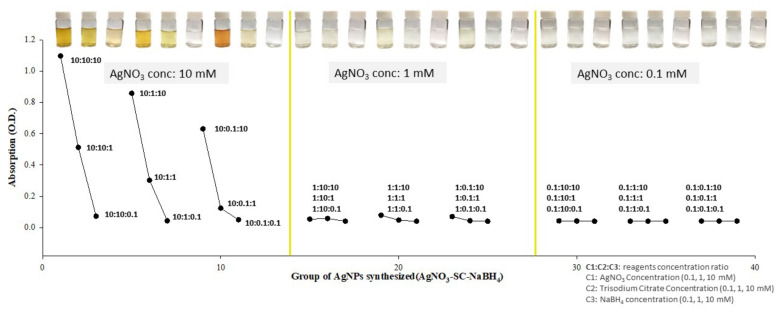
UV–Vis spectra of different concentrations at the wavelength of 400 nm for AgNPs generated using three reagents (silver nitrate, trisodium citrate, and sodium borohydride). Each dot represents a different reagent concentration ratio (silver nitrate/trisodium citrate/sodium borohydride); namely, 10:10:10, 10:10:1, 10:10:0.1, …, and 0.1:0.1:0.1.

**Figure 6:**
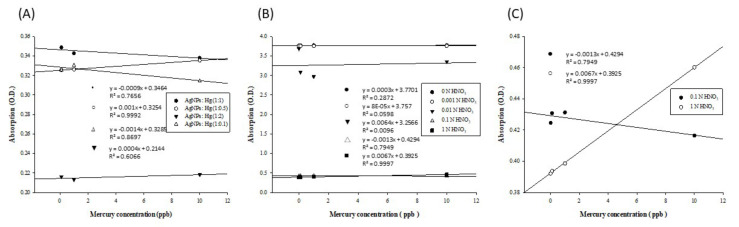
AgNP-based spectroscopic detection of mercury: (**A**) UV–Vis spectrum (at λ = 400 nm) for detection of mercury using silver nanoparticles in different reaction volume ratios. UV–Vis spectrum (at λ = 400 nm) for detection of mercury prepared with different nitric acid concentrations using silver nanoparticles; (**B**) three different concentrations of mercury prepared with five concentrations of nitric acid; and (**C**) mercury prepared with 0.1 and 1 N nitric acids. The 1 N nitric acid samples were the best of the five concentrations for diluted mercury standard solution for detection. The best R^2^ value in the curves was obtained when adding 1 N of nitric acid with the reagent.

The three reaction reagents were prepared at the concentrations of 10, 1, and 0.1 mM, and the absorbance values of the AgNPs synthesized with different concentration combinations were evaluated at a wavelength of 400 nm. The results are compared in Figure 5, where the concentration of the reactant increases from left to right. The higher the concentration of silver nitrate, the higher the concentration and content of the AgNP product, and the darker the orange color of the resulting solution. With the same silver nitrate and trisodium citrate concentrations, the concentration of the reducing agent can also affect the formation of AgNPs. As indicated in Figure 6, when the reagent concentration ratio is between 10:10:10 and 10:10:0.1, although the concentration of silver nitrate was high, the AgNP generation reaction was not completed with the addition of 0.1 mM sodium borohydride. Similarly, when the concentrations of silver nitrate and sodium borohydride were fixed, changing the concentration of the capping agent trisodium citrate decreased the proportion of AgNPs being capped; then, the AgNPs may aggregate, and the absorbance values will decrease accordingly. The AgNPs were also fabricated with high silver nitrate concentrations of 10, 1, and 0.1 M, and the resulting solutions had a brick red to gray cement color. These dark colors were caused by extremely high AgNP concentrations and more severe AgNP aggregation.

For the AgNPs generated using silver nitrate, trisodium citrate, and sodium borohydride, the concentration of the reactant mainly determined the final concentration of AgNPs. The content of the capping agent (i.e., trisodium citrate) can influence the size of the synthesized AgNPs [26]. Additionally, the concentration and volume of the reducing agent (sodium borohydride) can affect the completeness of the AgNP generation reaction and the initial form of the NPs.

### 3.3. AgNP-based Spectroscopic Detection of Mercury

To explore the optimal volume ratio of the reagents, we tested the volume ratios of the AgNP solution to the mercury standards (0.1/1/10 ppb) of 1:0.1, 1:0.5, 1:1, and 1:2. The linear curve in Figure 6A is based on measuring the absorbance of the reaction solution at 400 nm. Linearity is an evaluation criterion for the optimal volume ratio. Linearity with at least R^2^ > 0.99 can be achieved using a reagent volume ratio of 1:0.5. As the concentration of the mercury standard increased, the color generated by the reaction between AgNPs and mercury gradually changed from yellow to light yellow and, finally, transparent from the adsorption of mercury ions onto AgNPs to form amalgam crystals [31,44].

The mercury standards were prepared with different solvents and then tested with the AgNP solution for a coloring reaction. We prepared the mercury standards with 0, 0.001, 0.01, 0.1, and 1 N nitric acid solutions, conducted the coloring reaction with a volume ratio of AgNP solution to mercury standard of 1:0.5, and measured the absorbance values at a wavelength of 400 nm. The results are shown in Figure 6B. Among the mercury standards prepared with solvents at five different concentrations, the one dissolved in 1 N nitric acid exhibited the best linear trend (Figure 6C) when detected using AgNPs. Therefore, 1 N nitric acid was applied to prepare the mercury standards for the subsequent detection reactions and sample measurements.

#### Calibration Curve Construction for AgNP-Based Mercury Detection

**Figure 7:**
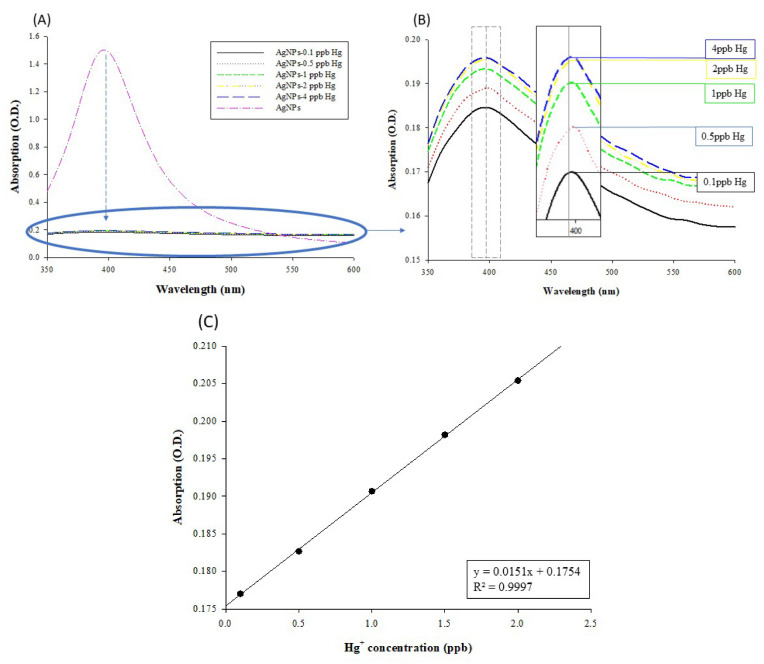
Establishment of the calibration curve for AgNP-based mercury detection: (**A**) UV–Vis absorption spectrum of the silver nanoparticles (AgNPs) and AgHg, the lower lines in the graph indicate AgNPs with different concentrations of mercury standard solutions; (**B**) UV–Vis absorption spectrum of AgNPs with different concentrations of mercury. Mercury concentration ranged from 0.1 to 4 ppb; and (**C**) UV–Vis spectrum at λ = 400 nm: standard curve for mercury detection using silver nanoparticles (AgNPs). Hg concentration ranged from 0.1 to 2 ppb. The regression was 0.99 (R^2^ > 0.99).

The results of AgNP-based spectroscopy for mercury detection are shown in Figure 7A. The absorbance values of the AgNPs not bound with mercury (the pink spectrum) exceeded 1.0. After binding the AgNPs with mercury to form amalgam crystals, the absorbance values declined and showed an upward trend in the low concentration range of 0.1–4 ppb (Figure 7B,C). Vasileva et al. reported that the relative intensity of the localized surface plasmon absorption band of AgNPs at 406 nm is linearly dependent on the concentration of Hg^2+^, with a positive slope for the concentration range 0–12.5 μg/L and a negative slope for the concentration range 25–500 μg/L [31]. This phenomenon is because the amalgam formed by mercury ions and AgNPs affects the surface of NPs no longer oxidized, so the detection range of Hg^2+^ is limited. Although the continuous improvement of the detection limit of AgNPs for Hg^2+^ was not affected [45,46,47,48,49,50], this meant that the color rendering methodology using the absorbance value of amalgam may correspond to two completely different Hg concentrations. Therefore, this study recommends that when applying the mercury contamination concentration range for the first time and/or at an unexpected detection range, carrying out a double or more dilution for the sample, and measuring with this approach, so the absorbance of this diluted sample will clearly show whether the concentration locates in the 0–12.5 μg/L or 25–500 μg/L range.

Metal nanoparticles have unique properties and applications in many fields, attributed to the collective dipole oscillation called surface plasmon resonance (SPR) [51]. This phenomenon makes them very suitable for the colorimetric sensing of Hg^2+^ ions. The interaction between the nanoparticles and the analyte changes the absorption band’s intensity and/or position in the visible spectrum, which can usually be observed by the naked eye [52]. The limitations of these systems are mainly related to poor selectivity, high detection limits of Hg^2+^, complex probe material synthesis, or complex analysis procedures. We used AgNPs to perform a stable colorimetric analysis of 0.1–2 ppb Hg^2+^ ions. Because of the redox interaction between AgNPs and Hg^2+^ ions, the absorbance intensity was expected to change. The Hg concentration determines which of the two redox reactions is dominant because the two oxidants compete for Ag oxidation. In this way, excess environmentally relevant mercury levels can be detected. Several sensing systems based on the interaction between AgNPs and Hg^2+^ ions have been reported [52,53]. However, in another competitive system, a detailed study of the behavior of mercury in the presence of an oxidant has rarely been carried out or discussed.

With high mercury concentrations, the amalgam formed by mercury ions and AgNPs coats the surface of AgNPs, such that the surface of the NPs is no longer oxidized, and the measured absorbance values tend to decrease because of the high refractive index [31]. Thus, referring to the regulatory detection limits of mercury, we established a calibration curve of mercury in the low concentration range (0.1–2 ppb), as shown in Figure 7C. With a linearity of R^2^ > 0.99, this curve can serve as a standard for subsequent sample measurements.

### 3.4. False-Positive Tests

**Figure 8:**
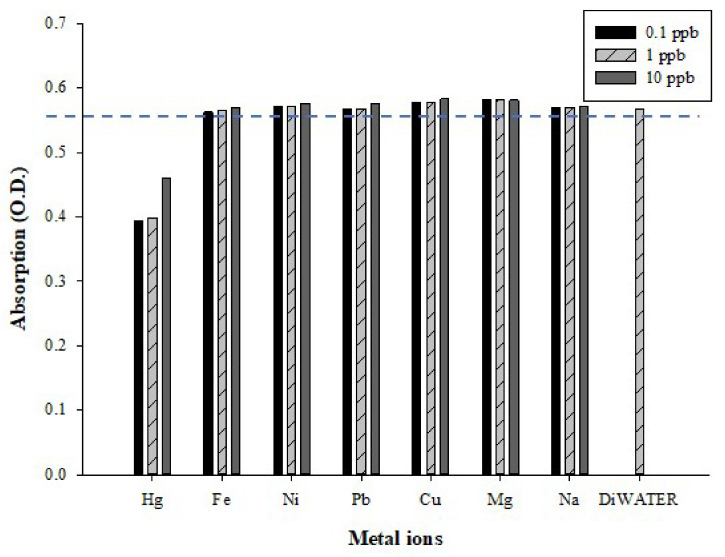
False-positive tests: UV–Vis spectra at a wavelength of 400 nm for AgNPs combined with different metal ions and deionized water (control). The metal ion concentrations were 0.1, 1, and 10 ppb.

Heavy metals discharged into the environment that cause pollution include not only mercury but also other metal ions. For the design of interference tests, we examined whether the AgNPs have false-positive test results with other metal ions. In our experiments, iron, nickel, lead, copper, magnesium, sodium, and mercury standards with a concentration of 0.1/1/10 ppb were used as analytes to react with the AgNPs. The results were compared with those of deionized water. The absorbance values were measured at a wavelength of 400 nm and plotted as a histogram, as shown in Figure 8. Considering the control group of deionized water as a reference standard, among the seven tested metal ions, only mercury reacted to the AgNPs. Other scholars have also conducted tests using the same interferents, and the metal ions with a concentration of 1.5–3 μM did not undergo a coloring reaction with AgNPs, except for mercury ions [54,55]. Based on the above analysis results, it can be concluded that the AgNPs synthesized by the chemical reduction method using trisodium citrate and sodium borohydride as the capping and reducing agents, respectively, can specifically detect mercury ions.

Regarding the UV–Vis spectrophotometer [31] and CVAAS [56], the mercury detection limits are approximately 0.1 ppb and 0.25 ppb, respectively. Therefore, the optimized AgNP color rendering range in this study is about 0.1–2 ppb, with great application potential. Taiwan’s national announcement limits the amount of mercury and methylmercury in different foods; for example, the mercury limit for packaged water and edible ice is 1 ppb, while the limit for salt is 100 ppb. In the future, we will use AgNPs as a coloring reagent to develop an excellent chemical coloration method; furthermore, this will be combined with appropriate detection modules in order to develop detection applications for mercury contamination in food.

### 3.5. Mercury Detection with Cold Vapor Atomic Absorption Spectroscopy

**Figure 9:**
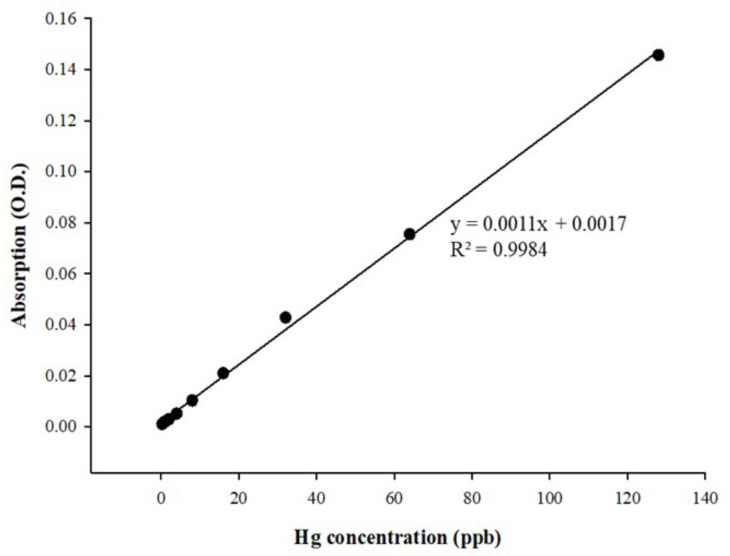
Linear calibration curve of mercury (Hg) obtained using atomic absorption spectroscopy (AAS) assay. The concentration of mercury (Hg) ranged from 0.25 to 128 ppb. The regression value was 0.9984 (R^2^ > 0.99).

We employed cold vapor atomic absorption spectroscopy [29] to determine the optimal mercury detection condition in this study; the constructed calibration curve is shown in Figure 9. The curve has good linearity in the concentration range of 0.25–128 ppb. In the literature, the methods of cold vapor atomic absorption spectroscopy and inductively coupled plasma atomic emission spectroscopy were compared, and their sample detection results showed no difference. Moreover, mercury detection with cold vapor atomic absorption spectroscopy is an easier task, without a tedious sample extraction procedure, and has a lower risk [26].

## 4. Conclusions

Currently, methods for detecting heavy metals in food mostly rely on advanced instruments, with fast-screening tests also being developed. However, most of the obtained results are semi-quantitative or qualitative. This study provided a successful application using silver nanoparticles in low concentration for rapid 0.1 ppb mercury detection. In addition, we successfully clarified the factors influencing the formation of AgNPs, including the reagent addition sequence, reaction temperature, reaction time, the pH of the solution, and the concentrations of the three reaction reagents. The optimal process for AgNP generation was as follows: Mix 10 mM of silver nitrate with trisodium citrate in a hot water bath for 10 min; add 10 mM of sodium borohydride to produce AgNPs and keep stirring for 2 h; then, adjust the pH to 12; finally, store the product in a low-temperature environment away from light.

Referring further to the detection limit of mercury in relevant governmental food regulations, we established a calibration curve of mercury in the concentration range of 0.1–2 ppb using AgNP-based spectroscopic measurement at a wavelength of 400 m, where the calibration curve exhibited good linearity (R^2^ > 0.99), demonstrating promising potential for the detection range in food. In addition, six metal ions—iron, nickel, lead, copper, magnesium, and sodium—were examined for false-positive tests and did not have a coloring reaction that interfered with the AgNP-based mercury detection. Since food ingredients are far more complex than wastewater, there are sample processing, decolorization, ashing, extraction, and sampling concentration range in mercury contamination inspection procedures. Therefore, the application of this research to the food field in the future needs further discussion. Overall, this research provides a stable and excellent AgNPs synthesis technology that will improve various AgNP-mediated reactions and food mercury contamination inspection.
